# A report on state‐wide implementation of newborn screening for X‐linked Adrenoleukodystrophy

**DOI:** 10.1002/ajmg.a.61171

**Published:** 2019-05-10

**Authors:** Katie Wiens, Susan A. Berry, Hyoung Choi, Amy Gaviglio, Ashish Gupta, Amy Hietala, Daniel Kenney‐Jung, Troy Lund, Weston Miller, Elizabeth I. Pierpont, Gerald Raymond, Holly Winslow, Heather A. Zierhut, Paul J. Orchard

**Affiliations:** ^1^ Division of Genetics and Metabolism, Departments of Pediatrics and Genetics, Cell Biology & Development University of Minnesota Minneapolis MN USA; ^2^ Division of Pediatric Neurology, Department of Neurology University of Minnesota Minneapolis MN USA; ^3^ Minnesota Department of Health St. Paul MN USA; ^4^ Division of Blood and Marrow Transplantation, Department of Pediatrics University of Minnesota Minneapolis MN USA; ^5^ Sangamo Therapeutics, Inc. Brisbane CA USA; ^6^ Division of Clinical Behavioral Neuroscience,Department of Pediatrics University of Minnesota Minneapolis MN USA; ^7^ Division of Pediatric Neurology, Department of Pediatrics Penn State Health Milton S. Hershey Medical Center Hershey PA USA; ^8^ Department of Genetics, Cell, Biology and Development University of Minnesota Minneapolis MN USA

**Keywords:** *ABCD1*, adrenal insufficiency, incidence, newborn screen, prevalence, X‐linked adrenoleukodystrophy

## Abstract

Minnesota became the fourth state to begin newborn screening (NBS) for X‐linked adrenoleukodystrophy (X‐ALD) in 2017. As there is limited retrospective data available on NBS for X‐ALD, we analyzed Minnesota's NBS results from the first year of screening. C26:0 lysophosphatidylcholine (C26:0‐LPC) screening results of 67,836 infants and confirmatory testing (*ABCD1* gene and serum VLCFA analysis) for screen positives were obtained. Fourteen infants (nine males, five females) screened positive for X‐ALD and all were subsequently confirmed to have X‐ALD, with zero false positives. The birth prevalence of X‐ALD in screened infants was 1 in 4,845 and 1 in 3,878 males, more than five times previous reported incidences. Pedigrees of affected infants were analyzed, and 17 male (mean age of 17) and 24 female relatives were subsequently diagnosed with X‐ALD. Phenotypes of these family members included self‐reported mild neuropathy symptoms in two males and seven females, and childhood cerebral disease (ccALD) and adrenal insufficiency in one male. We observed fewer cases of ccALD and adrenal insufficiency than expected in male family members (5.9% of males for both) compared to previous observations. Together, these findings suggest that the spectrum of X‐ALD may be broader than previously described and that milder cases may previously have been underrepresented. Other challenges included a high frequency of variants of uncertain significance in *ABCD1* and an inability to predict phenotypic severity. We posit that thoughtful planning to address these novel challenges and coordination by dedicated specialists will be imperative for successful implementation of population‐based screening for X‐ALD.

## 1 INTRODUCTION

X‐linked adrenoleukodystrophy (X‐ALD), is the most common peroxisomal disorder, with an estimated incidence of ~1 in 17,000 births (male and female) and 1 in 21,000 males in the United States (Bezman et al., 2001). X‐ALD is caused by mutations in the *ABCD1* gene, located on the X chromosome (Mosser et al., 1993). The *ABCD1* gene encodes the peroxisomal membrane protein, ATP‐binding cassette sub‐family D member one, also known as adrenoleukodystrophy protein, or ALDP (Mosser et al., 1993). ALDP transports very long chain fatty acids (VLCFA) from the cytosol into the peroxisome where the VLCFA are metabolized by beta‐oxidation. Pathogenic variants in the *ABCD1* gene lead to absent or abnormally functioning ALDP, resulting in an accumulation of VLCFA in plasma and tissues, including the brain, spinal cord, and adrenal cortex (Kemp & Wanders, 2010; Singh, Moser, Moser, & Kishimoto, 1984).

Clinical manifestations of X‐ALD are highly variable, even in the same family, and there is neither apparent correlation between genotype and phenotype, nor between degree of VLCFA accumulation and phenotype (Kemp, Berger, & Aubourg, 2012). The principle phenotypes observed in males with X‐ALD are adrenal insufficiency (Addison disease), adrenomyeloneuropathy (AMN), and cerebral ALD (Bezman & Moser, 1998). Affected males may manifest any combination of these, or in rare circumstances, none at all.

Most males with X‐ALD will develop primary adrenal insufficiency, marked by elevated adrenocorticotropic hormone (ACTH) and low levels of cortisol, typically in the first decade of life (Blevins, Shankroff, Moser, & Ladenson, 1994). Lifetime prevalence of adrenal insufficiency in males with X‐ALD is ~80% (Huffnagel et al., 2019). Adrenal crisis in individuals with undiagnosed adrenal insufficiency can result in rapid deterioration and death. Thus, early diagnosis of X‐ALD is vital as affected males with a previously unknown diagnosis are at increased risk for adrenal crisis with physiologic stressors such as surgery or illness (Raymond, Jones, & Moser, 2007).

The childhood cerebral form of the disease, marked by neuroinflammation, and demyelinating lesions in the cerebral white matter, occurs in ~35% of boys with X‐ALD (Mahmood, Dubey, Moser, & Moser, 2005). Early clinical manifestations of cerebral disease can be subtle, and include declining school performance, clumsiness, inattention, behavioral problems, and emotional lability. Progressive auditory and visual impairment may follow as the demyelination advances (Kemp et al., 2012; Kemp, Huffnagel, Linthorst, Wanders, & Engelen, 2016; Raymond et al., 2007). Evidence of disease is most commonly observed between ages 4 and 10 years, and progression is rapid; without treatment, cerebral disease leads to a vegetative state or death, on average, 2 years after the onset of symptoms (Kemp et al., 2012, 2016; Mahmood et al., 2005; Raymond et al., 2007). MRI abnormalities can be detected, on average, 2 years prior to the emergence of significant functional neurologic deficits (Mahmood et al., 2005; Moser et al., 2005).

Males who do not develop childhood cerebral disease will, in most circumstances, develop AMN later in life. AMN is an axonopathy of the spinal cord, with onset most commonly in the third or fourth decade (Engelen et al., 2012). Symptoms include spastic paraparesis, sphincter disturbances, and sexual dysfunction. Severity of disease is highly variable, with some men requiring the use of wheelchairs, while others have only very minor deficits even into their 60s or 70s (Turk, Moser, & Fatemi, 2017).

Woman who are X‐ALD carriers (i.e., heterozygous for an *ABCD1* pathogenic variant) do not typically develop cerebral disease or adrenal insufficiency (Engelen et al., 2014). However, up to 88% of women may become symptomatic with a myelopathy or peripheral neuropathy in adulthood (Engelen et al., 2014). Symptoms are typically milder than those observed in males with AMN and with a later age of onset.

Standard treatment for cerebral ALD is HSCT. When successful, HSCT leads to stabilization of white matter lesions around 6–12 months after engraftment, without any reversal or repair of the already established lesions (Kemp et al., 2016; Miller et al., 2011; Shapiro et al., 2000). However, as there is significant morbidity and mortality associated with this treatment, HSCT is only recommended for individuals with evidence of active cerebral disease (Kemp et al., 2016; Raymond et al., 2007). Five‐year survival after HSCT in those with minimal cerebral involvement (Loes score < 10 and/or no clinical evidence of neurologic dysfunction) is 91% (Miller et al., 2011). When performed at a later stage of disease (Loes score ≥ 10), mortality is increased (62% 5‐year survival for boys with clinical evidence of neurologic dysfunction at time of HSCT), and HSCT may not be recommended at all if disease is exceptionally progressed (Mahmood, Raymond, Dubey, Peters, & Moser, 2007; Miller et al., 2011; Peters et al., 2004; Raymond et al., 2007; Shapiro et al., 2000).

New York became the first state to add X‐ALD to their state's newborn screening program on December 30, 2013 (Vogel et al., 2015). Prior to newborn screening, only males who were diagnosed at a young age with X‐ALD because of a known family history or because of etiologic work‐up of clinical primary adrenal insufficiency had the opportunity for routine screening with brain MRI for cerebral disease. As white matter abnormalities on brain imaging precede outward neurological manifestations, there is a window of opportunity to intervene for individuals with early, clinically pre‐symptomatic, cerebral disease.

With New York's experience and because of the potential for improvement in outcome with early diagnosis, ongoing monitoring, and timely treatment, X‐ALD was added to the Federal recommended uniform screening panel (RUSP) in February 2016. States individually choose if and how to implement screening for each disorder, resulting in various screening models and implementation timelines across the country. However, in general, newborn screening for X‐ALD is accomplished by examining C26:0‐lysophosphatidylcholine (C26:0‐LPC) values in dried blood spots collected as part of routine newborn screening. NBS programs may choose to further supplement this analysis with *ABCD1* sequencing on any specimen with elevation of C26:0‐LPC.

Minnesota became the fourth state to begin universal newborn screening for X‐ALD on February 6, 2017. In this article, we report outcomes from the first year of screening for X‐ALD through the state's newborn screening program and highlight the public health implications of adding this X‐linked condition with variable presentation to newborn screening programs. The goal of the study was to ascertain birth prevalence and phenotypic presentation of X‐ALD among screened infants and subsequently diagnosed family members, and to identify key downstream considerations including issues pertaining to subsequent family member diagnoses and the identification of variants of uncertain significance in the *ABCD1* gene.

## 2 METHODOLOGY

A retrospective review of Minnesota Department of Health (MDH) C26:0‐LPC newborn screening results and associated follow‐up outcomes from the first year of screening was completed.

In Minnesota, screening for X‐ALD is completed by measuring C26:0‐LPC in dried blood spots using negative ion‐mode liquid chromatography tandem mass spectrometry (LC‐MS/MS) (Haynes & De Jesús, 2012). Samples with a C26:0‐LPC value <0.16 μmol/L are considered screen negative and no further action is required. Samples with a C26:0‐LPC value ≥0.30 μmol/L are considered screen positive and the patient is referred for further testing and diagnostic evaluation. Samples with a C26:0‐LPC value between 0.16–0.29 μmol/L are considered screen borderline, and a repeat heel‐stick specimen is requested. If this repeat sample is again ≥0.16 μmol/L, the infant is considered screen positive. If the repeat sample is <0.16 μmol/L, the case is considered resolved and no further action is recommended.

The total number of infants screened and their C26:0‐LPC results (screen positive, screen borderline, and screen negative) were assessed to determine the observed frequency of positive screens in males and females during the first year of implementation of the assay. Additionally, C26:0‐LPC values for positive screens were analyzed.

Upon a positive screen, Minnesota Department of Health staff contacted the primary care provider for the infant, and the recommendation was made to consult with one of three genetics centers in the state. All but two infants who screened positive for this assay during the period analyzed were ultimately evaluated at the University of Minnesota Medical Center (UMMC).

All families seen at UMMC received genetic counseling and a consultation with a pediatric neurologist as part of their initial visit. Confirmatory serum VLCFA analysis and *ABCD1* mutation analysis were obtained at an initial visit, if not already performed prior to the visit. Clinical history was obtained and neurologic evaluation of the infant completed. Additionally, a family history (minimum three generation pedigree) was taken and reviewed to assess for at‐risk family members. Families were encouraged to communicate risk information to family members, and information regarding appropriate testing for those family members at risk (VLCFA analysis and/or *ABCD1* mutation analysis) was provided. Pedigrees were updated as family members completed testing.

In cases where a variant of uncertain significance (VUS) was identified in the *ABCD1* gene, targeted mutation analysis of other family members was completed to determine whether the variant tracked with biochemical disease (elevated VLCFA) in an attempt to clarify the significance of the variant.

*ABCD1* variants, VLCFA results, and phenotypes of infants who screened positive on the C26:0‐LPC assay were obtained and reviewed as part of routine follow‐up by the Minnesota NBS program and University of Minnesota, following IRB approval. Additionally, pedigrees were analyzed to assess the total number of affected family members and frequency of reported phenotypes for those who were found to have X‐ALD (both hemizygous males and heterozygous females).

## 3 RESULTS

### 3.1 Birth prevalence of X‐ALD and efficacy of C26:0‐LPC for detection of disease

**Figure 1:**
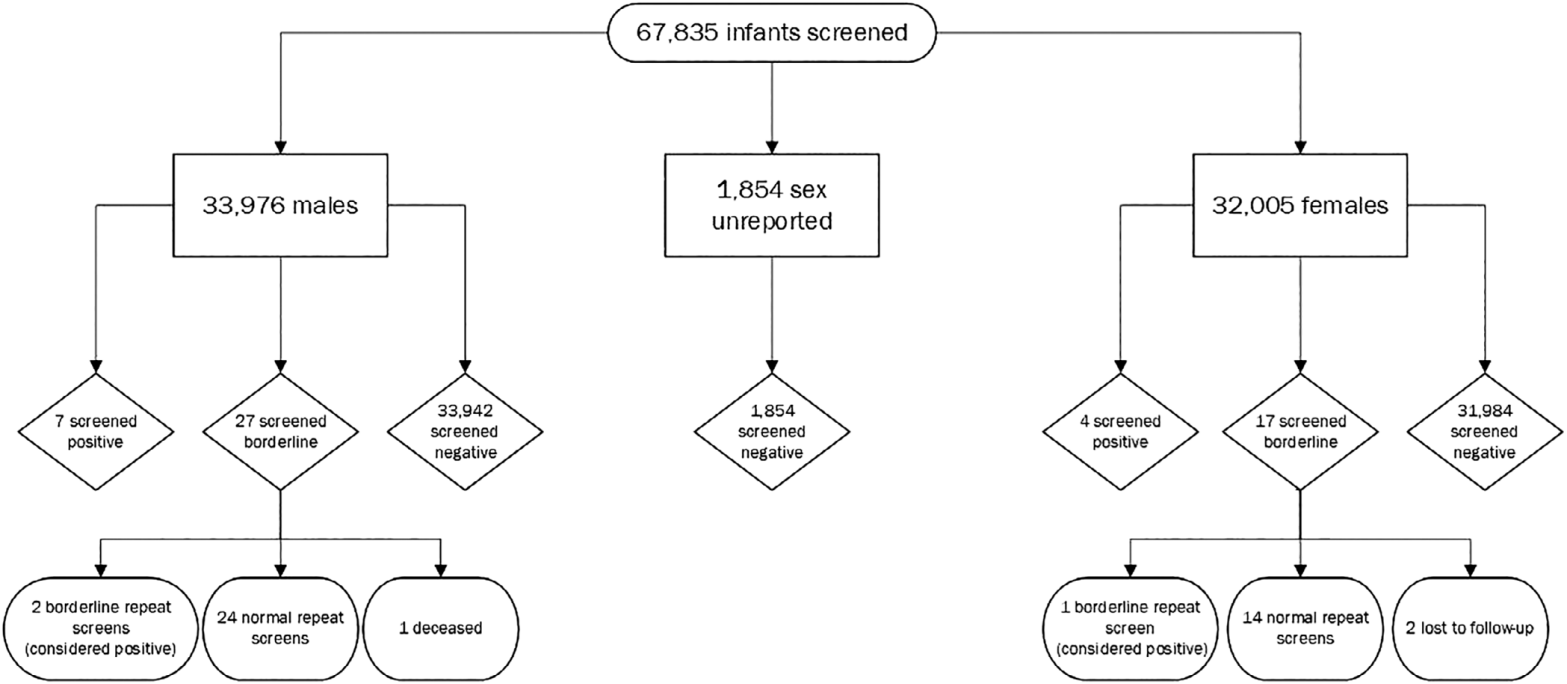
Flowchart demonstrating results of infants screened for ALD on NBS during Minnesota's first year of screening. ALD, adrenoleukodystrophy; NBS, newborn screening

In the first year of screening, 67,835 infants were screened in Minnesota, including 33,976 males, 32,005 females, and 1,854 cases where sex was not reported on the screening card *(*Figure 1). Of all infants screened, 11 screened positive (seven males, four females), while 44 screened borderline (27 males, 17 females). Of those that screened borderline, the requested repeat screen was deemed positive for three infants (two males, one female), for a total of 14 screen positive results. Two female borderline cases were lost to follow‐up, and one male died prior to a repeat screen. To the best of our knowledge, this death was unrelated to a possible diagnosis of X‐ALD or other peroxisomal disorder.

All infants who screened positive for X‐ALD via C26:0‐LPC on NBS were subsequently confirmed positive by serum VLCFA analysis. This confirmatory analysis was performed at one of two clinical laboratories, Kennedy Krieger Institute or Mayo Medical Labs, with different units of measurement and cut‐off values utilized at each laboratory. VLCFA results were converted to μmol/L to allow for comparison between laboratories (Table 1). Given that all infants were subsequently confirmed positive with diagnostic testing, there were zero false positive results for X‐ALD during the time analyzed; positive predictive value (PPV) for screen positive cases = 100%. Of the 44 infants with initial screen borderline results, the majority were within normal limits on a separate repeat specimen; however, three were screen positive on their repeat specimen and were subsequently confirmed to have X‐ALD via serum VLCFA analysis (PPV for screen borderline cases = 6.8%).

**Table 1:** C26:0‐LPC, serum C26:0, *ABCD1* variant and classification, inheritance and family history for positive screens

Case	NBS C26:0 LPC (μmol/L)	VLCFA C26:0	*ABCD1* variant	Classification by clinical laboratory	Previously reported	Inherited	Known family history
F1^a^	0.17	2.14 μmol/L	c.508G > A (p.Ala170Thr)	Variant of uncertain significance	Yes	Unknown	No
F2	0.54	2.09 μmol/L	c.1635‐16_1645delins CACAGACATGTAGGGC	Likely pathogenic	No	Maternal	No
F3	0.57	1.84 μmol/L	c.1978C > T (p.Arg660Trp)	Pathogenic	Yes	De novo	No
F4^b^	0.3	Declined	N/A	N/A	N/A	Maternal	Yes
F5	0.64	2.65 μmol/L	c.1553G > A (p.Arg518Gln)	Pathogenic	Yes	Maternal	Yes
M1^a^	0.26	3.34 μmol/L	No variant detected	N/A	N/A	Maternal	No
M2^a^	0.16	1.53 μmol/L	c.80A > C (p.Tyr27Ser)	Likely Pathogenic^c^	No	Maternal	No
M3	0.72	10.60 μmol/L	c.293C > T (p.Ser98Leu)	Pathogenic	Yes	Presumed maternal^d^	No
M4	0.35	3.18 μmol/L	c.823C > T (p.Arg275Trp)	Likely Pathogenic^c^	No	Maternal	No
M5	0.76	6.90 μmol/L	c.487C > T (p.Arg163Cys)	Likely pathogenic	No	Presumed Maternal^d^	No
M6	0.4	2.17 μmol/L	c.1597A > C (p.Lys533Gln)	Likely pathogenic	Yes	Maternal	No
M7	0.47	N/A	c.823C > T (p.Arg275Trp)	Likely Pathogenic^c^	No	Maternal	No
M8	0.43	1.92 μmol/L	c.593C > T (p.Thr198Met)	Likely pathogenic	Yes	Maternal	No
M9	1.12	8.067 μmol/L	c.1973C > T (p.Thr658Ile)	Likely pathogenic	Yes	Unknown	No

Abbreviations: C26:0‐LPC, C26:0 lysophosphatidylcholine; VLCFA, very long chain fatty acids; X‐ALD, X‐linked adrenoleukodystrophy.

a Cases where repeat screen after borderline result was abnormal. This is the C26:0‐LPC value of the final repeat screen.

b This female infant had a known family history of X‐ALD. Prenatal testing via amniocentesis revealed the familial *ABCD1* variant; however, parents declined genetics follow‐up after birth, and the Minnesota Department of Health was not notified of specific disease‐causing *ABCD1* variant.

c Initially classified as a variant of uncertain significance.

d Presumed maternal based on two sons with X‐ALD; however, confirmatory testing in mother not completed.

*ABCD1* analysis was also completed in all cases, and a variant was detected in all but one case (Table 1). This case is discussed in further detail below.

The false negative rate was not specifically assessed in the current study. No reports of false negative cases were received during the study period, though the time period analyzed may be too short for these cases to have presented clinically.

Based on results from the first year of screening in Minnesota, the overall birth prevalence of X‐ALD in this population was 1 in 4,845. Prevalence in males was estimated to be 1 in 3,878 (nine affected males/33,976 total male infants +927 [half of unknown gender cases]). Female prevalence was slighter lower, estimated at 1 in 6,586 (five affected females/32,005 total female infants +927 [half of unknown gender cases]). This lower prevalence could indicate that some female heterozygotes are missed by the NBS assay, which would corroborate previous work showing that female heterozygotes typically have lower C26:0 values than affected hemizygous males, and thus may have screening values under the cut‐off range.

### 3.2 VLCFA results, *ABCD1* variants and phenotypes of proband

All 14 confirmed X‐ALD infants were asymptomatic at initial consultation. Specifically, none had hypotonia, seizures, feeding difficulties, or any abnormalities on neurologic or physician exam that could indicate a different peroxisomal disorder. Adrenal function studies as well as brain MRIs performed at 1 year of age have been normal and without evidence of cerebral disease in all infants who had these additional screening studies after diagnosis.

Assessment of confirmatory serum C26:0 values was performed. Values for females ranged from 1.84 to 2.65 μmol/L with an average of 2.18 μmol/L. Values for males ranged from 1.53 to 10.60 μmol/L with an average of 4.71 μmol/L. (Table 1).

Of the 14 positive newborn screening cases, *ABCD1* variant analysis results were available for all but one case at the time of this review. This individual, F4, had a known family history of X‐ALD, and prenatal amniocentesis reportedly identified that this female carried the familial *ABCD1* variant. As such, parents declined follow‐up with genetics and postnatal confirmatory testing after positive newborn screen.

Another individual (M1) did not have an identifiable *ABCD1* gene variant by next generation sequencing and deletion/duplication analysis. This individual's older brother, mother, and maternal male first cousin were also found to have elevated VLCFA. There was no family history of cerebral disease or adrenal insufficiency; however, his maternal grandmother endorses pain in her feet and difficulty walking, especially when going up stairs, since her mid‐50s. Based on the family history suggestive of X‐linked inheritance, and the fact that all affected individuals are asymptomatic, other autosomal recessive peroxisomal disorders were not considered. As a result, only *ABCD1* analysis was completed. Functional studies on the patient's fibroblasts to assess for adrenoleukodystrophy protein (ALDP) was subsequently sent, and returned with significantly reduced presence ALDP on immunoblot, further confirming the diagnosis of X‐ALD for this individual.

In the other 12 cases, initial *ABCD1* gene analysis demonstrated three pathogenic variants, five likely pathogenic variants, and four variants of uncertain significance (Table 1). Seven of the variants were previously reported, and five were novel.

Four infants' (F1, M2, M4, M7) *ABCD1* analyses initially returned with a variant of uncertain significance. Two of these infants had the same *ABCD1* variant but were seemingly unrelated. After targeted variant analysis in other affected and unaffected family members (based on VLCFA results; most family members were clinically asymptomatic) and showing that the variant tracked with biochemical results in these family members, all but one of these variants of uncertain significance was subsequently reclassified as likely pathogenic by the performing clinical diagnostics laboratory. The one that was not reclassified was one of the individuals not seen at UMMC, and thus it is unclear if segregation studies were performed.

Two male infants (M3 and M9) had VLCFA values that were far more elevated than is typical in X‐ALD, 10.60 and 8.07 μmol/L, respectively. While both had VLCFA analysis completed at different diagnostic laboratories, M9 had VLCFA analysis in a laboratory reporting C26:0 value in the reference range typical of that seen in individuals with Zellweger syndrome (reported mean for X‐ALD hemizygote = 3.28 μmol/L; mean for Zellweger syndrome = 9.91 μmol/L). C26:0‐LPC on dried blood spot for M9 was also 1.12 μmol/L, which was the highest value of all the screen positive cases, with the next highest being 0.76 μmol/L. Because of these significantly elevated VLCFA values, a peroxisomal gene panel was recommended by the clinical teams of both infants rather than simply analyzing *ABCD1*. Plasmalogens, pipecolic acid, phytanic acid, and pristanic acid were also sent for M9 and were normal. Results of genetic testing returned with a pathogenic *ABCD1* variant for both M3 and M9, and no other variants were detected in any of the other analyzed genes.

### 3.3 Family member analysis

Of the 14 probands, family history and subsequent familial diagnostic workup was available for 13. Two of these 13 probands had a known family history of X‐ALD prior to the proband's diagnosis on NBS, and for the purpose of this report, these two cases were not included in further familial analysis. For the remaining 11 families, 32 male family members had testing for ALD (primarily VLCFA analysis) following the proband's diagnosis, and 17 of these males had elevated VLCFA, consistent with X‐ALD (Table 2). Additionally, 24 females were confirmed to be a carrier of X‐ALD, either through confirmatory testing (VLCFA analysis and/or *ABCD1* targeted mutation analysis) or because they were an obligate carrier based on the family history. Thus, a total of 41 family members were diagnosed with X‐ALD (either hemizygote or heterozygote) following diagnosis in a proband on NBS, for an average of 3.7 family member diagnoses per proband. Importantly, not all at‐risk family members had completed testing at the time of this article, indicating this number is likely an underestimate of the true number of affected family members that will be diagnosed as a result of newborn screening.

**Table 2:** Demographics and phenotypes of ALD in family members

Number of diagnosed family members	41
Males	17
Females	24
Age of diagnosed male family members	
Range	1–67 years
Average	17 years
Median	10 years
Phenotypes of family members	
Males with cerebral disease	1 (5.9% of males)
Males with adrenal insufficiency	1 (5.9% of males)
Males with AMN symptoms	2 (11.8% of all males, 66.7% of males >18 years)
Females with AMN symptoms	7 (29.2%)

*Note*: Demographic and phenotypic information for family members diagnosed with X‐ALD following diagnosis in proband. This data excludes information from two families with a known family history of X‐ALD prior to diagnosis in proband.

Abbreviations: ALD, adrenoleukodystrophy; AMN, adrenomyeloneuropathy; X‐ALD, X‐linked adrenoleukodystrophy.

Only one case was confirmed to have occurred de novo, for a de novo rate of around 7.7%. An additional family had not completed targeted maternal testing at the time of the study.

At the time of this writing, aside from the clinical findings in families with a known family history of X‐ALD prior to newborn screening, mild neuropathy symptoms were self‐reported in 11.8% of male family members (66.7% of males >18 years of age), and 29.2% of female family members (Table 2). Only one of the subsequently diagnosed males were found to have childhood cerebral disease. Thus, the frequency of cerebral disease found after first evaluation of affected male family members was 5.9%. However, it must be noted that aside from this symptomatic male, 7 of the other 16 male relatives with X‐ALD were younger than 10 years of age, and thus still at a high risk for developing childhood cerebral disease. The individual with active cerebral disease had no outward clinical symptoms of cerebral disease; however, brain MRI revealed T2 hyperintensity within the white matter and corpus callosum. His cerebral disease was caught relatively early, with a Loes score of two, and hematopoietic stem cell transplantation was recommended. This same male was also found to have adrenal insufficiency.

## 4 DISCUSSION

This is one of the first reports describing outcomes of population screening of X‐ALD on a newborn screening platform. While the benefits of adding X‐ALD to newborn screening programs and the potential lives saved are undeniable, there are several novel challenges not previously encountered with other conditions tested for in NBS programs.

Our study found that over the first year of screening in Minnesota, 14 infants screened positive for X‐ALD on NBS, and all were subsequently confirmed positive. These results suggest that C26:0‐LPC detection by LC‐MS/MS is an effective and specific population‐based screening assay for X‐ALD, with a high positive predictive value with confirmation of all screen positive results during the year analyzed. As five females were also detected, this NBS assay may also be a reliable test for detecting female heterozygotes. In fact, one study concluded that C26:0‐LPC from dried blood spots is actually more sensitive at detecting female heterozygotes than serum VLCFA levels (Huffnagel et al., 2017).

### 4.1 Variability in prevalence of X‐ALD

In the first year of screening in Minnesota, the birth prevalence of X‐ALD in males was found to be 1 in 3,878. This birth prevalence is more than five times the incidence in males observed by Bezman et al. (2001) in which the incidence was calculated based on VLCFA results from two of the main laboratories that completed testing at the time, Kennedy Krieger Institute and Mayo Medical Laboratories (Bezman et al., 2001). The birth prevalence found in Minnesota was also more than four times that observed in males detected by NBS in New York State, which was 1 in 16,074 (Caggana, 2018). The overall birth prevalence in both males and females in Minnesota was 1 in 4,845, which was comparable, though still higher, to that detected by NBS in Connecticut, which was 1 in 9,341.

One factor contributing to the observed higher birth prevalence in Minnesota may simply be the result of the population‐based screening approach undertaken with NBS. Individuals who may not have otherwise come to medical attention due to mild phenotypes are identified and diagnosed in the newborn period. This theory would infer that there are hundreds of males who have X‐ALD, but are clinically asymptomatic, or have previously escaped medical detection, and thus are underrepresented in past birth prevalence data. This would further suggest a broader spectrum of disease, with more males falling into the mild or asymptomatic categories than previously reported. However, this hypothesis does not explain why the birth prevalence of X‐ALD on NBS in New York is four times less frequent than Minnesota's observed birth prevalence.

Another potential explanation for the variable frequencies in NBS populations across states could be differences in cut‐off values or screen borderline/ screen positive categories, which are determined by each state independently. This could mean that states with lower reported frequencies are potentially missing cases due to utilization of higher cut‐off values. Alternatively, states with more conservative cut‐off values, such as Minnesota, are potentially identifying false‐positive cases. The latter seems less likely, as all positive screens in Minnesota were confirmed to have X‐ALD.

Another explanation could simply be a higher incidence of X‐ALD in the state of Minnesota for reasons that are currently unclear. It is also possible that Minnesota had a skewed birth prevalence in the first year of screening, and that the true birth prevalence will decline over time. However, to fully appreciate the causes of the frequency differences, more time will be needed to both assess clinical symptomatology of the infants identified and determine if cases are being missed, as no false‐negative results have been reported thus far, even in states with higher cut of values.

### 4.2 Phenotypic spectrum

Disease presentation for X‐ALD is highly variable, even within the same family. This means that once a newborn is diagnosed, we are not able to predict in which way, at which age, or how severely, the disease will present in that individual.

To date, all newborns detected by newborn screening in Minnesota have been asymptomatic without adrenal or cerebral involvement. This is not surprising, as the oldest individual identified by NBS is only around 24 months of age at the time of this writing, younger than we would typically expect signs or symptoms to manifest. It is expected that with time, some of these boys will be diagnosed with primary adrenal insufficiency and childhood cerebral disease.

Eleven probands without prior known family history of X‐ALD received care at the University of Minnesota. From these 11 probands, an additional 41 family members (17 male and 24 females) were confirmed to also have X‐ALD. Of these family members, cerebral disease was subsequently diagnosed in one individual, a 6‐year‐old brother of the proband identified on NBS. This same individual also had adrenal insufficiency. All other affected males had brain MRI and adrenal function studies that were normal.

AMN‐like symptoms were self‐reported in two males and seven females. Worth noting, the majority of adult family members did not have a neurologic evaluation, so it is possible AMN symptoms would have been evident on exam in individuals that self‐reported no consistent clinical manifestations.

These mild/asymptomatic phenotypes in affected family members, in combination with the higher observed prevalence of disease, raises the possibility that the spectrum of ALD may be broader than previously thought, and that milder/seemingly asymptomatic cases could have previously been underrepresented.

### 4.3 Genotypic spectrum

Both frameshift and missense variants in the *ABCD1* gene were detected, and there were no apparent mutational hotspots. There were numerous novel variants which appeared to be private, familial variants.

Four variants were initially classified as a variant of uncertain significance, three of which were subsequently reclassified as likely pathogenic after the variants were found to co‐segregate with elevated VLCFA in the family. This highlights the importance of completing follow‐up familial studies to aid in interpretation of variants of uncertain significance. If familial testing is not an option, or if the variant remains a VUS after these studies, functional screening on fibroblasts for ALDP expression may prove beneficial in ruling in or out a diagnosis of X‐ALD. However, this testing is not readily available in the US at present. Reclassification of variants of uncertain significance is particularly important when families are planning on using this genetic information for reproductive purposes in future pregnancies.

Furthermore, this emphasizes the importance of contributing genotypic information to variant databases, such as the ALD Mutation Database (2019) (“ALD Mutation Database”). As there are hundreds of different pathogenic variants in *ABCD1*, many of which are novel or seemingly unique to a family, it is important to share variant information in order to aid in determining the pathogenicity of variants if seen in the future.

### 4.4 Spectrum of diagnostic results

Much is often learned about the phenotypic spectrum of disease after population‐based screening such as NBS, and X‐ALD is likely to be no exception. In addition to the higher prevalence than previously reported with a more prominent mild/asymptomatic phenotype, the cases below highlight that there is still much to be learned to regard the diagnostic tools used for X‐ALD (genetic testing and VLCFA analysis).

One male (M1) had a personal and family history (brother, mother, and maternal male cousin) of elevated VLCFA; however, no *ABCD1* variant was detected by next generation sequencing or deletion/duplication analysis. Functional studies on the patient's fibroblasts returned with only trace ALDP on immunoblot, confirming the diagnosis of X‐ALD for this individual. While an *ABCD1* variant has been detected in all previously published cases of X‐ALD, one author (G. Raymond) has previously cared for a single family with X‐ALD without an identifiable *ABCD1* mutation. It is possible that these families have a variant in *ABCD1* that cannot be detected by standard sequencing technology, such as an intronic variant or structural rearrangement. Therefore, it may prove true that a diagnosis of X‐ALD cannot be definitively excluded in cases without a detectable *ABCD1* variant based on standard sequencing methods, especially when a family history is suggestive of an X‐linked inheritance pattern. In these scenarios, functional screening on fibroblasts for ALDP expression may prove beneficial in ruling in or out a diagnosis of X‐ALD.

Two males (M3 and M9) had confirmatory VLCFA values that were far more elevated than is typical in X‐ALD, and were more in line with values seen in other, more severe, peroxisomal conditions. A differential diagnosis to include other peroxisomal disorders (such as Zellweger syndrome) was therefore considered and a peroxisomal gene panel was recommended rather than *ABCD1* analysis alone. For both males, genetic testing returned with a likely pathogenic variant in the *ABCD1* gene, and no other variants were detected in any of the other genes analyzed. Therefore, the diagnosis of X‐ALD was confirmed based on genotypic information for these individuals. These cases highlight the limitation of using VLCFA values alone as a diagnostic tool in a proband, and the importance of follow‐up genetic testing to differentiate between various peroxisomal conditions, especially in the absence of clinical symptoms.

### 4.5 Impact of X‐linked disorders after NBS

X‐ALD is the first condition on the RUSP with X‐linked inheritance; prior disorders have been conditions inherited in an autosomal recessive pattern. Typically, with autosomal recessive inheritance, only siblings of an affected individual are at risk to also have the condition. In contrast, for disorders with autosomal dominant and X‐linked inheritance patterns, numerous family members in different generations can be affected.

Based on a de novo rate of only 7.7% in our study, and a similar rate in other studies, it is clear that X‐ALD is inherited in the vast majority of cases (Wang et al., 2011). Once a proband is diagnosed on NBS, there are often dozens of family members that are at‐risk for the condition. Because of the highly variable nature of the disease presentation even within a family, a diagnosis cannot be ruled out simply because someone is seemingly healthy without associated symptoms. These family members should undergo diagnostic testing to determine whether they have X‐ALD.

Logistically, it can be difficult to have family members complete diagnostic testing. In UMMC's experience, around half of primary care providers felt comfortable ordering VLCFA for their patients; however, no primary care providers felt comfortable ordering targeted *ABCD1* analysis. While this is understandable given the complexities of molecular testing, result interpretation, and test cost/health‐insurance coverage, this means numerous at‐risk family members did not get tested or had to travel to a specialty center offering genetic counseling to coordinate testing. The high number of at‐risk family members recommended being tested also corresponded to increased workload for our center in trying to help coordinate diagnostic testing for family members. This also led to increased referrals once family members had confirmatory testing and were found to have X‐ALD, or referrals if a family member could not have testing done locally and wanted to pursue testing at UMMC.

This is not different from if an individual is diagnosed following symptoms of childhood cerebral disease or primary adrenal insufficiency, but the coordination and follow up move from clinical centers to the newborn screening programs and their participating genetic centers. This diagnostic domino effect is something not seen with the other conditions on NBS and should be taken into consideration to ensure appropriate resources are available when adding X‐ALD to a state's NBS program.

### 4.6 Conclusion & future directions

The benefits of adding X‐ALD to newborn screening programs and the potential lives saved are predicted to be significant. The LC‐MS/MS assay used in Minnesota has proven to be robust and highly effective at detecting affected individuals, with Minnesota confirming all screen positive results in the first year of screening. However, there are unique challenges with adding this condition to NBS including variants of uncertain significance, inability to predict phenotypic severity in confirmed cases, and downstream diagnoses of numerous family members based on the X‐linked inheritance pattern.

Based on these unexpected challenges, our center adjusted protocols for handling NBS cases, both in regard to initial notification and confirmatory testing, and as it relates to ongoing care for this patient population.

Initially, following notification of a positive NBS for X‐ALD, the primary care provider was requested to order confirmatory VLCFA analysis, and only refer if X‐ALD was confirmed. Because this process took over a month in numerous cases, and taking into consideration the reliability of the NBS assay for this condition, the protocol was updated to eliminate the need for the primary care provider to order confirmatory testing. Rather, patients were immediately referred to a genetic counselor where the condition was discussed, family history obtained to assess for other at‐risk males, and confirmatory VLCFA and *ABCD1* analysis drawn. This allowed families to receive accurate information and confirmation of diagnosis in a timely manner.

In an attempt to meet the ongoing needs of this large patient population, a multidisciplinary adrenoleukodystrophy clinic was created at UMMC to meet the comprehensive medical needs of pre‐symptomatic newborns detected by NBS and their family members.

Specialty centers in states that are just beginning screening for X‐ALD may want to proactively assign responsibility for the initial notification and follow‐up diagnostic testing for these cases. Given the high positive predictive value of the LC‐MS/MS screening assay for X‐ALD, a different approach may need to be considered for this condition compared to other screened conditions which have higher rates of false positive screens. Furthermore, states should be prepared for, and anticipate detecting, far more cases than predicted based on previously reported disease incidence and may want to proactively consider where the medical home for these patients will be.

Overall, adding X‐ALD to Minnesota's newborn screening program proved immediately beneficial. Not only were a high number of probands detected and placed on a monitoring protocol, but a number of affected family members were also subsequently detected, including one young male with early, clinically‐silent cerebral disease, and adrenal insufficiency. X‐ALD was much more common than anticipated with a birth prevalence of 1 in 4,845 in the first year of screening. With the higher anticipated birthprevalence and subsequent detection and diagnosis of numerous family members, thoughtful planning and coordination by specialists will be imperative for successful implementation of population‐based screening for X‐ALD.

## CONFLICT OF INTEREST

Heather Zierhut is on the Board of Directors of GeneMatters, LLC, a remote telehealth genetic counseling company. Weston Miller is a full time employee at Sangamo Therapeutics.

## Data Availability

The data that support the findings of this study are available from the corresponding author upon reasonable request.

## References

[ajmga61171-bib-0001] ALD Mutation Database . (2019). Retrieved March 6, 2019, from https://adrenoleukodystrophy.info/mutations-and-variants-in-abcd1

[ajmga61171-bib-0002] Bezman, L. , Moser, A. B. , Raymond, G. V. , Rinaldo, P. , Watkins, P. A. , Smith, K. D. , … Moser, H. W. (2001). Adrenoleukodystrophy: Incidence, new mutation rate, and results of extended family screening. Annals of Neurology, 49(4), 512–517. 10.1002/ana.101 11310629

[ajmga61171-bib-0003] Bezman, L. , & Moser, H. W. (1998). Incidence of X‐linked adrenoleukodystrophy and the relative frequency of its phenotypes. American Journal of Medical Genetics, 76, 415–419. 9556301

[ajmga61171-bib-0004] Blevins, L. S. , Shankroff, J. , Moser, H. W. , & Ladenson, P. W. (1994). Elevated plasma adrenocorticotropin concentration as evidence of limited adrenocortical reserve in patients with adrenomyeloneuropathy. Journal of Clinical Endocrinology and Metabolism, 78(2), 261–265. 10.1210/jcem.78.2.8106609 8106609

[ajmga61171-bib-0005] Caggana, M . (2018). Update on X‐ALD screening in the New York Laboratory. Retrieved January 8, 2018, from https://www.aidanhasaposse.org/uploads/7/3/6/5/73650801/new_york_ald_nbs_update_michele_caggana_sc.d._facmg.pdf

[ajmga61171-bib-0007] Engelen, M. , Barbier, M. , Dijkstra, I. M. E. , Schür, R. , de Bie, R. M. A. , Verhamme, C. , … Kemp, S. (2014). X‐linked adrenoleukodystrophy in women: A cross‐sectional cohort study. Brain, 137(3), 693–706. 10.1093/brain/awt361 24480483

[ajmga61171-bib-0008] Engelen, M. , Kemp, S. , De Visser, M. , Van Geel, B. M. , Wanders, R. J. A. , Aubourg, P. , & Poll‐The, B. T. (2012). X‐linked adrenoleukodystrophy (X‐ALD): Clinical presentation and guidelines for diagnosis, follow‐up and management. Orphanet Journal of Rare Diseases, 7, 51 10.1186/1750-1172-7-51 22889154 PMC3503704

[ajmga61171-bib-0009] Haynes, C. A. , & De Jesús, V. R. (2012). Improved analysis of C26:0‐lysophosphatidylcholine in dried‐blood spots via negative ion mode HPLC‐ESI‐MS/MS for X‐linked adrenoleukodystrophy newborn screening. Clinica Chimica Acta, 413(15–16), 1217–1221. 10.1016/j.cca.2012.03.026 22503909

[ajmga61171-bib-0010] Huffnagel, I. C. , Laheji, F. K. , Aziz‐Bose, R. , Tritos, N. A. , Marino, R. , Linthorst, G. E. , … Eichler . (2019). The natural history of adrenal insufficiency in X‐linked Adrenoleukodystrophy: An international collaboration. Journal of Clinical Endocrinology and Metabolism, 104(1), 118–126. 30252065 10.1210/jc.2018-01307

[ajmga61171-bib-0011] Huffnagel, I. C. , van de Beek, M. C. , Showers, A. L. , Orsini, J. J. , Klouwer, F. C. C. , Dijkstra, I. M. E. , … Kemp, S. (2017). Comparison of C26:0‐carnitine and C26:0‐lysophosphatidylcholine as diagnostic markers in dried blood spots from newborns and patients with adrenoleukodystrophy. Molecular Genetics and Metabolism, 122(4), 209–215. 10.1016/j.ymgme.2017.10.012 29089175

[ajmga61171-bib-0012] Kemp, S. , Berger, J. , & Aubourg, P. (2012). X‐linked adrenoleukodystrophy: Clinical, metabolic, genetic and pathophysiological aspects. Biochimica et Biophysica Acta, Molecular Basis of Disease, 1822, 1465–1474. 10.1016/j.bbadis.2012.03.012 22483867

[ajmga61171-bib-0013] Kemp, S. , Huffnagel, I. C. , Linthorst, G. E. , Wanders, R. J. , & Engelen, M. (2016). Adrenoleukodystrophy ‐ Neuroendocrine pathogenesis and redefinition of natural history. Nature Reviews. Endocrinology, 12, 606–615. 10.1038/nrendo.2016.90 27312864

[ajmga61171-bib-0014] Kemp, S. , & Wanders, R. (2010). Biochemical aspects of X‐linked adrenoleukodystrophy Brain Pathology (Vol. 20, pp. 831–837). 10.1111/j.1750-3639.2010.00391.x 20626744 PMC8094824

[ajmga61171-bib-0016] Mahmood, A. , Dubey, P. , Moser, H. W. , & Moser, A. (2005). X‐linked adrenoleukodystrophy: Therapeutic approaches to distinct phenotypes. Pediatric Transplantation, 9 (Suppl 7), 55–62. 10.1111/j.1399-3046.2005.00447.x 16305618

[ajmga61171-bib-0017] Mahmood, A. , Raymond, G. V. , Dubey, P. , Peters, C. , & Moser, H. W. (2007). Survival analysis of haematopoietic cell transplantation for childhood cerebral X‐linked adrenoleukodystrophy: A comparison study. Lancet Neurology, 6(8), 687–692. 10.1016/S1474-4422(07)70177-1 17618834

[ajmga61171-bib-0018] Miller, W. P. , Rothman, S. M. , Nascene, D. , Kivisto, T. , Defor, T. E. , Ziegler, R. S. , … Orchard, P. J. (2011). Outcomes after allogeneic hematopoietic cell transplantation for childhood cerebral adrenoleukodystrophy: The largest single‐institution cohort report. Blood, 118(7), 1971–1978. 10.1182/blood-2011-01-329235 21586746

[ajmga61171-bib-0019] Moser, H. W. , Raymond, G. V. , Lu, S. E. , Muenz, L. R. , Moser, A. B. , Xu, J. , … Odone, A. (2005). Follow‐up of 89 asymptomatic patients with adrenoleukodystrophy treated with Lorenzo's oil. Archives of Neurology, 62, 1073–1080. 10.1001/archneur.62.7.1073 16009761

[ajmga61171-bib-0020] Mosser, J. , Douar, A. M. , Sarde, C.‐O. , Kioschis, P. , Feil, R. , Moser, H. , … Aubourg, P. (1993). Putative X‐linked adrenoleukodystrophy gene shares unexpected homology with ABC transporters. Nature, 361(6414), 726–730. 10.1038/361726a0 8441467

[ajmga61171-bib-0021] Peters, C. , Charnas, L. R. , Tan, Y. , Ziegler, R. S. , Shapiro, E. G. , DeFor, T. , … Krivit, W. (2004). Cerebral X‐linked adrenoleukodystrophy: The international hematopoietic cell transplantation experience from 1982 to 1999. Blood, 104(3), 881–888. 10.1182/blood-2003-10-3402 15073029

[ajmga61171-bib-0022] Raymond, G. V. , Jones, R. O. , & Moser, A. B. (2007). Newborn screening for adrenoleukodystrophy: Implications for therapy. Molecular Diagnosis & Therapy, 11, 381–384. 10.1007/BF03256261 18078355

[ajmga61171-bib-0023] Shapiro, E. , Krivit, W. , Lockman, L. , Jambaqué, I. , Peters, C. , Cowan, M. , … Aubourg, P. (2000). Long‐term effect of bone‐marrow transplantation for childhood‐onset cerebral X‐linked adrenoleukodystrophy. Lancet, 356(9231), 713–718. 10.1016/S0140-6736(00)02629-5 11085690

[ajmga61171-bib-0024] Singh, I. , Moser, A. E. , Moser, H. W. , & Kishimoto, Y. (1984). Adrenoleukodystrophy: Impaired oxidation of very long chain fatty acids in white blood cells, cultured skin fibroblasts, and amniocytes. Pediatric Research, 18(3), 286–290. 10.1203/00006450-198403000-00016 6728562

[ajmga61171-bib-0025] Turk, B. R. , Moser, A. B. , & Fatemi, A. (2017). Therapeutic strategies in adrenoleukodystrophy. Wiener Medizinische Wochenschrift, 167(9–10), 219–226. 10.1007/s10354-016-0534-2 28493141

[ajmga61171-bib-0026] Vogel, B. H. , Bradley, S. E. , Adams, D. J. , D'Aco, K. , Erbe, R. W. , Fong, C. , … Caggana, M. (2015). Newborn screening for X‐linked adrenoleukodystrophy in New York state: Diagnostic protocol, surveillance protocol and treatment guidelines. Molecular Genetics and Metabolism, 114(4), 599–603. 10.1016/j.ymgme.2015.02.002 25724074

[ajmga61171-bib-0027] Wang, Y. , Busin, R. , Reeves, C. , Bezman, L. , Raymond, G. , Toomer, C. J. , … Steinberg, S. (2011). X‐linked adrenoleukodystrophy: ABCD1 de novo mutations and mosaicism. Molecular Genetics and Metabolism, 104, 160–166. 10.1016/j.ymgme.2011.05.016 21700483

